# Low Ambient Temperature and Intracerebral Hemorrhage: The INTERACT2 Study

**DOI:** 10.1371/journal.pone.0149040

**Published:** 2016-02-09

**Authors:** Danni Zheng, Hisatomi Arima, Shoichiro Sato, Antonio Gasparrini, Emma Heeley, Candice Delcourt, Serigne Lo, Yining Huang, Jiguang Wang, Christian Stapf, Thompson Robinson, Pablo Lavados, John Chalmers, Craig S. Anderson, INTERACT2 investigators

**Affiliations:** 1 Division of Neurology and Mental health, The George Institute for Global Health, Sydney, New South Wales, Australia; 2 Sydney Medical School, the University of Sydney, Sydney, New South Wales, Australia; 3 Centre for Epidemiologic Research in Asia, Shiga University of Medical Sciences, Shiga University, Shiga, Japan; 4 Department of Social and Environmental Health Research, London School of Hygiene & Tropical Medicine, London, United Kingdom; 5 Royal Prince Alfred Hospital, Camperdown, New South Wales, Australia; 6 Department of Neurology, Peking University First Hospital, Beijing, China; 7 Centre for Epidemiological Studies and Clinical Trials, The Shanghai Institute of Hypertension, Ruijin Hospital, Shanghai Jiaotong University School of Medicine, Shanghai, China; 8 Department of Neurology, APHP–Hôpital Lariboisière, DHU NeuroVasc Paris–Sorbonne, Université Paris Diderot–Sorbonne Paris Cité, Paris, France; 9 Department of Neuroscience, CRCHUM, University of Montreal, Montreal, Quebec, Canada; 10 Department of Cardiovascular Sciences and NIHR Biomedical Research Unit in Cardiovascular Disease, University of Leicester, Leicester, United Kingdom; 11 Vascular Neurology Program, Neurology Services, Department of Internal Medicine, Clínica Alemana de Santiago, Universidad del Desarrollo, Santiago, Chile; Massachusetts General Hospital, UNITED STATES

## Abstract

**Background:**

Rates of acute intracerebral hemorrhage (ICH) increase in winter months but the magnitude of risk is unknown. We aimed to quantify the association of ambient temperature with the risk of ICH in the Intensive Blood Pressure Reduction in Acute Cerebral Haemorrhage Trial (INTERACT2) participants on an hourly timescale.

**Methods:**

INTERACT2 was an international, open, blinded endpoint, randomized controlled trial of patients with spontaneous ICH (<6h of onset) and elevated systolic blood pressure (SBP, 150–220 mmHg) assigned to intensive (target SBP <140 mmHg) or guideline-recommended (SBP <180 mmHg) BP treatment. We linked individual level hourly temperature to baseline data of 1997 participants, and performed case-crossover analyses using a distributed lag non-linear model with 24h lag period to assess the association of ambient temperature and risk of ICH. Results were presented as overall cumulative odds ratios (ORs) and 95% CI.

**Results:**

Low ambient temperature (≤10°C) was associated with increased risks of ICH: overall cumulative OR was 1.37 (0.99–1.91) for 10°C, 1.92 (1.31–2.81) for 0°C, 3.13 (1.89–5.19) for -10°C, and 5.76 (2.30–14.42) for -20°C, as compared with a reference temperature of 20°C.There was no clear relation of low temperature beyond three hours after exposure. Results were consistent in sensitivity analyses.

**Conclusions:**

Exposure to low ambient temperature within several hours increases the risk of ICH.

**Trial Registration:**

ClinicalTrials.gov NCT00716079

## Introduction

Acute stroke due to spontaneous (non-traumatic) intracerebral hemorrhage (ICH) is a major global health issue which causes death and permanent disability in several million people worldwide each year [1]. While marked seasonal and temporal patterns to the occurrence of ICH are recognised, with peak incidence in the winter and an association with cold temperature [2–5], this information is primarily derived from daily meteorological data without consideration of ambient temperature close to the time of onset of ICH. Moreover, the time lag method, which has shown an association of ambient temperature with acute myocardial infarction [6,7], has not been used in the study of ICH where influences on fluctuations in blood pressure (BP) appear especially important [8]. Thus, the association of ambient temperature with ICH onset has not been well quantified on an hourly time-scale. Such information could help our understanding of the determinants of ICH in high risk populations that have marked seasonal and geographical trends in rates [9], and guide public health strategies to optimize preventative strategies in winter.

The primary aim of this study was to quantify the transient increase in risk of ICH associated with declining ambient temperature at an hourly resolution. Secondary aims were to determine the time sequence of low temperature triggering effects on ICH occurrence and whether there is any variation in risks across several pre-defined patient characteristics.

## Materials and Methods

### Ethics Statement

The INTERACT 2 study protocol was approved by the ethics committees for each centers and written informed consent was obtained from all patients or relevant surrogates. A full list of centers that participated in the trial is shown in Acknowledgments. Patient records/information was anonymized and de-identified prior to analysis.

### Study Design

We conducted post-hoc analysis using the baseline patient data from the second (main phase) Intensive Blood Pressure Reduction in Acute Cerebral Haemorrhage Trial (INTERACT2). The design and main results of INTERACT2 have been described elsewhere.[10,11]. In brief, INTERACT2 was an international, multicenter, open, blinded endpoint, randomized controlled trial [12]. A total of 2839 patients with CT-confirmed spontaneous ICH, elevated systolic BP (SBP, 150-220mmHg), and capacity to commence BP lowering treatment (<6h of onset assessed by ‘time last seen normal’), were enrolled from 144 hospitals in 21 countries between 7th October 2008 and 30^th^ August 2012. Patients were excluded if they had a structural cerebral cause for the ICH, deep coma (defined as a score of 3–5 on the Glasgow coma scale [GCS] [13], where scores range from 3 to 15, with lower scores indicating reduced levels of consciousness), massive hematoma with a poor prognosis, or if early surgery to evacuate the hematoma was planned. Eligible patients were randomly assigned to receive intensive (target SBP <140mmHg within 1h) or guideline recommended (SBP <180mmHg) BP treatment. In order to minimize assessor bias, the primary outcome of ‘death or major disability’ (as measured by the modified Rankin scale) at 90 days was evaluated by clinicians who were blind to the randomized treatment. The date of the final patient follow up was 31 December 2012.

We obtained hourly ambient temperature data primarily from Weatherbank Inc (Edmond, US) which directly sources weather observations from worldwide government agencies that represent the official source of data in different countries. Data from the Australian Bureau of Meteorology was also used. For the main analyses, we linked baseline data of 1997 individual patients (70%) to hourly temperature data from monitoring stations less than 100km from the patients’ hospital of enrolment.

### Statistical Analyses

Baseline characteristics of the included patients were compared to those excluded in models using chi-square or Wilcoxon rank sum tests. We applied a time-stratified, case-crossover analysis to the data with the preceding 24h period of the reported ICH onset time selected as the case period for each ICH event [14–16]. Control periods of exposure were defined as the comparable 24h before the ICH in the other weeks of the same month and year. This method of selecting control periods has been shown to effectively control for time invariant confounders and seasonality, whilst also avoiding the problems of time trend and overlap bias associated with other methods of referent selection [16,17]. Thus, each participant was represented with a matched set of data for 1 case period and 3–4 control periods.

We assessed the risk of ICH in a distributed lag non-linear model (DLNM) which was originally developed to estimate the non-linear and delayed effects of temperature (or air pollution) on mortality or morbidity [18,19]. We applied recent developments in the method that extended DLNMs beyond time series data, and made it applicable in a case-crossover study design [20]. The DLNM involves a bi-dimensional space of functions that describes simultaneously the shape of the relationship along both the space of the predictor and the lag dimension of its occurrence. We modeled the exposure-response function with a natural cubic spline with internal knots placed at quartiles of ambient temperature and a reference temperature of 20°C (taken as the average optimal temperature of comfort for humans) [21], and the lag-response function was modeled with a natural cubic spline with two equally spaced internal knots in log-scale. A bi-dimensional plot was constructed to demonstrate the entire relationship between temperature and ICH risk whilst also taking into account the time sequence of effect. Lag-response curves were plotted to describe the evolving temporal change in ICH risk in a 24 h lag period after exposure to specific temperatures in comparison to a reference optimal temperature of 20°C. Furthermore, the effect of temperature was summarized in an overall cumulative exposure-response figure which shows the net increased ICH risk over an entire lag period of 24 h in association with temperature exposure, accounting for any harvesting or lagged effects. Finally, our results were also presented as overall cumulative odds ratios (ORs) and 95% confidence intervals (CI) that were computed by summation of the lag-specific risk contributions for a given pattern of temperature exposures during the 24 hours lag period. The statistical equation describing the DLNM model and derivation of the overall cumulative odds ratio is outlined elsewhere [20].

In order to check the robustness of the study results, several sensitivity analyses were performed; by inclusion of all patients with known hourly temperature data within 200km of the site of enrolment, which totalled 2346 (83%); and by extending the maximum lag length from 24 to 72 h. All analyses were performed using SAS 9.2 (SAS Institute Inc., Cary, NC) and R project for statistical computing V.3.1.0 using the ‘dlnm’ and ‘survival’ packages.

## Results

Table 1 shows the baseline demographic and clinical characteristics of patients with available ambient temperature data (‘included patients’, n = 1997) and those without such data (‘excluded patients’, n = 832). Those without this data were significantly younger, had less prior use of antithrombotic therapy, higher diastolic BP, and more likely to have lobar ICH and larger hematoma volumes. The weather parameters of the 79 cities included in analyses are outlined in the S1 Table.

**Table 1:** Patient characteristics.

Variable	Included patients (n = 1997)	Excluded patients (n = 832)	*p* value
**Time from ICH onset to randomization, median (IQR), h:min**	3:38 (2:45–4:40)	3:58 (3:00–4:40)	<0.001
**Age, mean (SD), y**	65 (13)	61 (12)	<0.001
**Male**	1252 (63)	528 (63)	0.70
**Medical history**			
Hypertension	1441 (72)	607 (73)	0.57
Previous ICH	150 (8)	79 (10)	0.07
Ischemic stroke	196 (10)	90 (11)	0.40
Diabetes mellitus	227 (11)	78 (9)	0.13
**Drug use**			
Antihypertensive therapy	170 (9)	107 (13)	<0.001
Antiplatelet therapy	225 (11)	40 (5)	<0.001
Warfarin anticoagulation	75 (4)	6 (1)	<0.001
**Clinical features**			
SBP, mean (SD), mmHg	179 (17)	180 (17)	0.13
DBP, mean (SD), mmHg	100 (15)	104 (13)	<0.001
Heart rate, mean(SD), bpm	78 (14)	78 (14)	0.22
NIHSS score,^a^ median (IQR)	10 (6–16)	11 (7–16)	0.31
GCS score,^b^ median (IQR)	14 (13–15)	14 (12–15)	<0.001
**ICH location**			0.01
Lobar	213 (11)	47 (7)	
Deep	1569 (83)	601 (84)	
Brainstem	52 (3)	28 (4)	
Cerebellum	58 (3)	31 (4)	
**Baseline ICH volume, median (IQR), mL**	10.6 (5.5–19.1)	11.8 (6.7–20.1)	0.01
**Intensive BP treatment**	989 (50)	410 (49)	0.91

Data are n (%), mean (SD), or median (IQR).

Comparisons for continuous and categorical variables were made using Wilcoxon and chi-square tests respectively.

ICH indicates intracerebral hemorrhage; SBP

systolic blood pressure; DBP, diastolic blood pressure; NIHSS, National Institute of Health stroke scale; GCS, Glasgow coma scale; BP, blood pressure.

^a^NIHSS can range from 0 (normal, no neurological deficit) to 42 (coma with quadriplegia).

^b^GCS scores can range from 3 (deep coma) to 15 (normal, alert)

**Fig 1:**
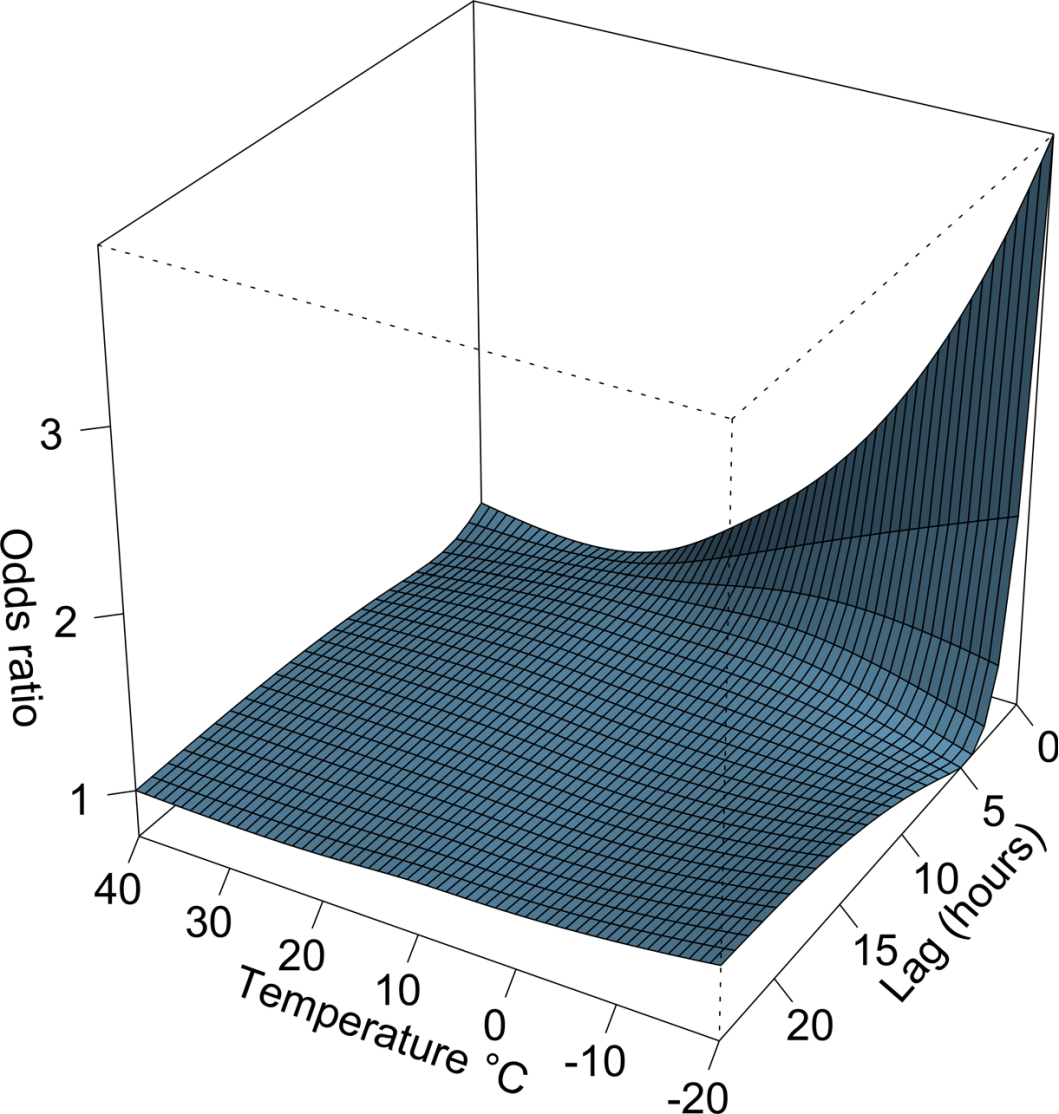
Bi-dimensional exposure-lag-response plot of intracerebral hemorrhage risk. The exposure-response function was modeled with a natural cubic spline with internal knots placed at quartiles of ambient temperature (and a reference temperature of 20°C), and the lag-response function was modeled with a natural cubic spline with two equally spaced internal knots in log-scale.

**Fig 2:**
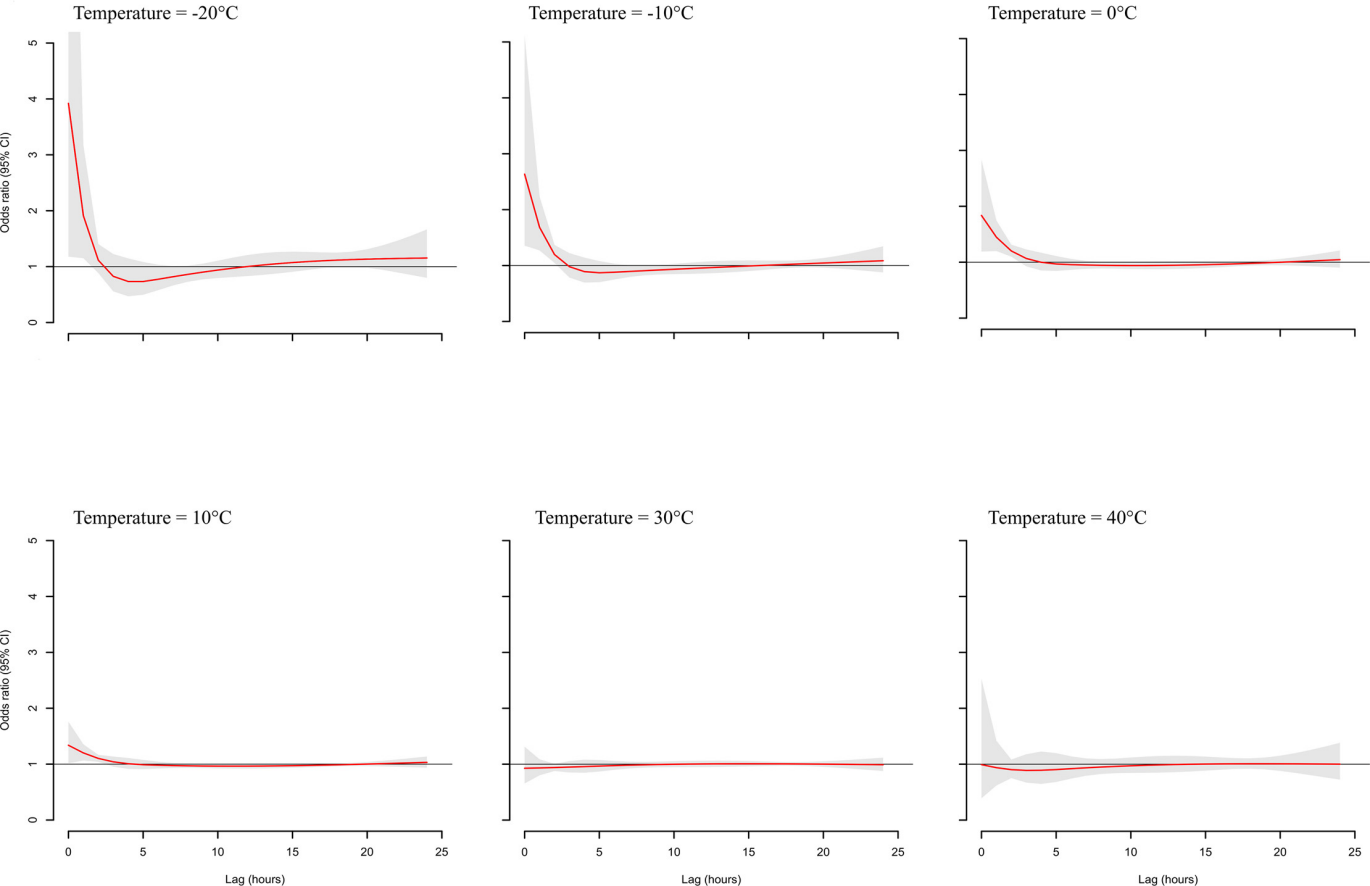
Lag-exposure plots of intracerebral hemorrhage risk for specific temperatures. CI indicates confidence interval. Case (n) indicates the number of ICH events in each temperature exposure category.

**Fig 3:**
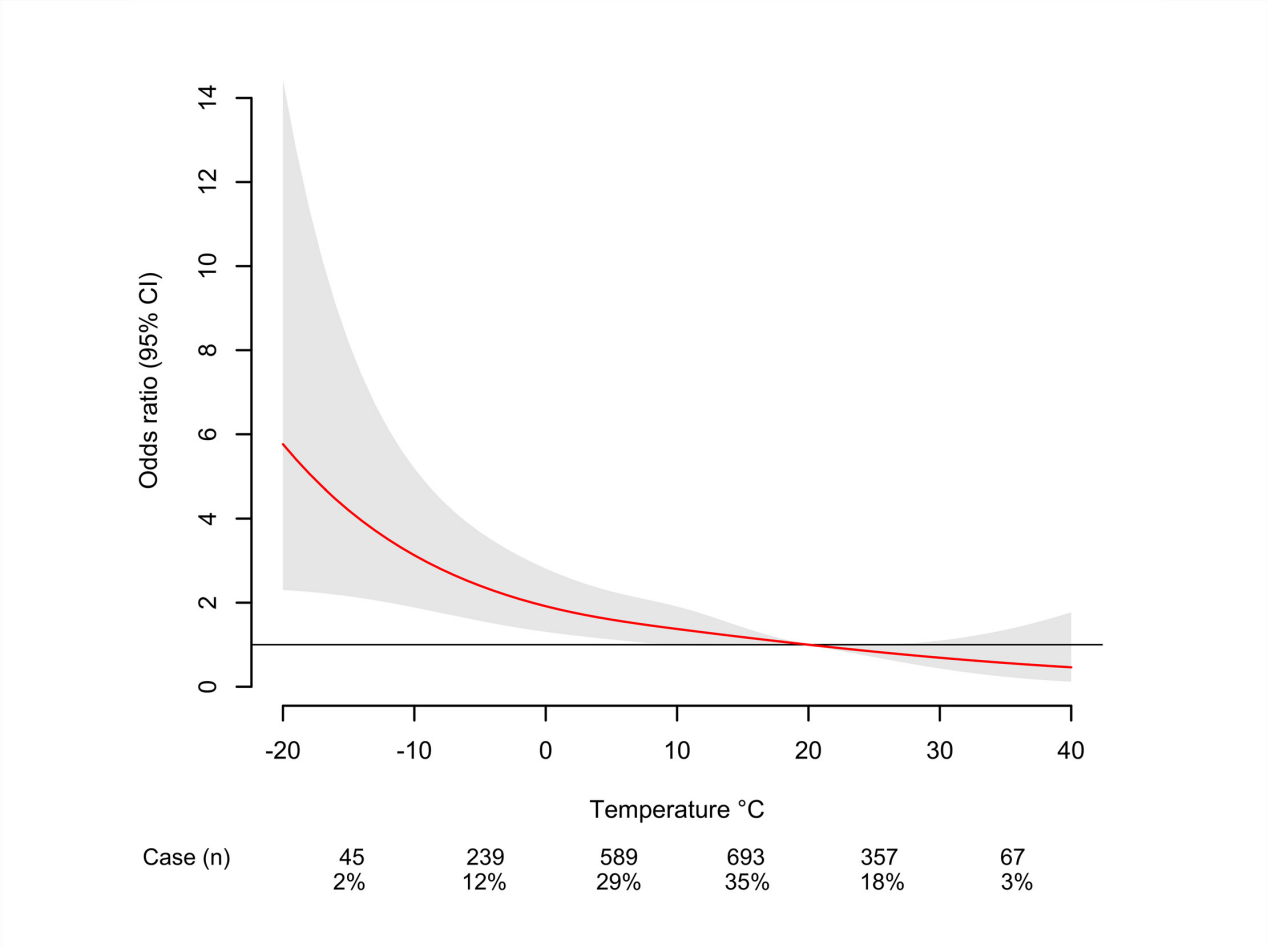
Overall cumulative odds ratio plot of temperature effect.

Fig 1 shows the bi-dimensional exposure-lag-response surface, depicting changes across temperature and lags with a trend of higher ORs at low ambient temperatures and shorter time lags. The lag-exposure plots in Fig 2 indicates an elevated risk of ICH in association with exposure to low ambient temperature (≤10°C) between lag 0 to lag 3 and that a harvesting effect seems to exist at longer lags. The main results of our study are shown in Fig 3, which reflects the relationship between low temperature exposure and ICH risk. A total number of 1568 (79%) ICH cases occurred below the optimal reference temperature. The overall cumulative ORs for ICH progressively increased from 1.37 (0.99–1.91) for 10°C, 1.92 (1.31–2.81) for 0°C, 3.13 (1.89–5.19) for -10°C, and 5.76 (2.30–14.42) for -20°C.

**Fig 4:**
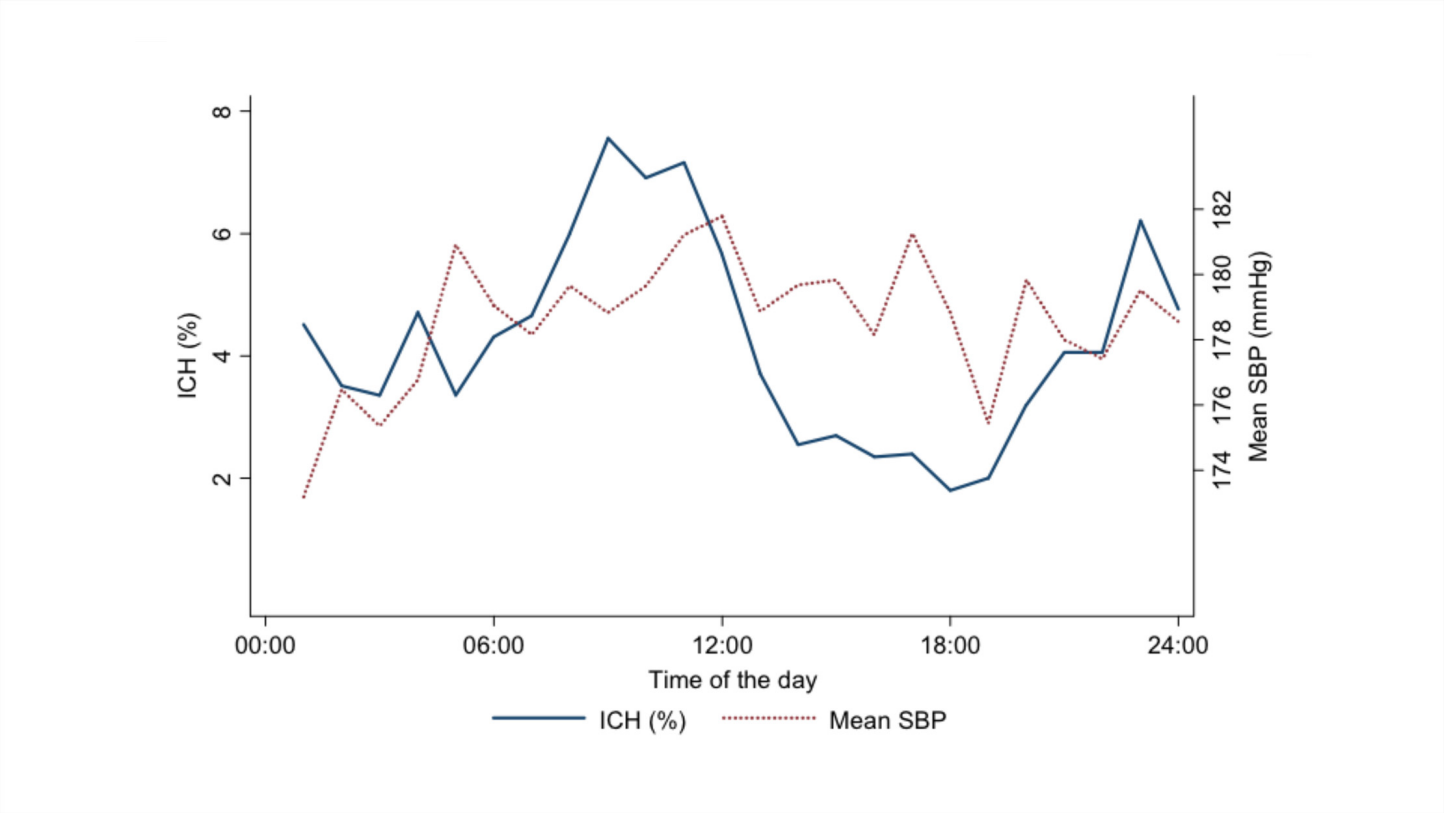
Temporal pattern of intracerebral hemorrhage onset and baseline systolic blood pressure level in patients. ICH indicates intracerebral hemorrhage; SBP, systolic blood pressure.

Fig 4 shows a temporal pattern to the occurrence of ICH, with peak frequencies in the early morning (06:00–10:00) and evening (20:00–23:00), but no clear relationship with the level of SBP among patients at the time of entry into the trial.

Results of sensitivity analyses including all patients with known hourly temperature data within 200km of the site of enrolment and extending the maximum lag length from 24 to 72h all produced similar results (S1–S3 Figs).

## Discussion

To our knowledge, this is the first large-scale international study to have investigated the association between ambient temperature and ICH risk at an hourly resolution and to capture the time sequence of the detrimental effect of low ambient temperature. Using distributed lag non-linear model fitted to a case-crossover model involving 1997 well-characterized ICH patients, we found a clear association of low ambient temperature with ICH occurrence within a few hours of exposure.

Several but not all studies have reported an association between low ambient temperature and ICH [2–5,22–24]. This discrepancy may be due to the use of daily temperature parameters that may not be truly representative of the temperature at the time of ICH onset. Furthermore, previous studies involved subjects from single regions, some of which have relatively small variations in temperature throughout the year. Our study using hourly temperature data in a large international study population corroborates the findings of a Taiwanese study with known hourly temperature data at ICH onset which showed a trend of increased ICH occurrence towards the lower temperature and is also consistent with prior investigations and clinical observation of a peak ICH occurrence in the winter due in part to the trigger effect of low ambient temperature on the risk of ICH onset at particular times of the day [2–5,25]. Mechanisms underlying the increased ICH risk in colder climate have not been resolved but exposure to low temperatures has been shown to stress the sympathetic nervous system leading to acute increases in heart rate, peripheral resistance, and thereby BP [26,27]. Although acute increases in BP associated with exposure to low ambient temperature is the most plausible trigger of ICH, we were unable to find any correlation between admission SBP level and ambient temperature, nor any differences in risks between those with and without a history of hypertension. This could be because the admission systolic BP of participants may not be representative of their BP level close to the time of ICH onset, or that ICH patients with non-elevated BP were excluded from our study.

Previous studies have reported upon a temperature effect for up to 14 days for the occurrence of myocardial infarction [6,7]. However, our study results show no association of low temperature beyond several hours prior to ICH onset and this finding is further supported by sensitivity analysis with extended lag period of 72 h. The differences in these risk time periods may be attributable to the distinctions in pathological mechanisms of onset for myocardial infarction (being plaque rupture from atherothrombosis) and ICH (being mainly due to the rupture of small deep perforating intracerebral vessels).

Key strengths of this study include the large sample of a wide range of patients with early and rigorous standardised evaluations of clinical and imaging findings after acute ICH. Also we were able to attain reliable hourly temperature weather data and link this information to the baseline patient characteristics to conduct precise evaluations on the effects of low ambient temperature. Our case-crossover model with control periods stratified by calendar month inherently adjusted for potential confounders, such as slow time varying individual characteristics, time of the day, day of the week, and seasonality. Furthermore, we were also able to avoid problems of time trend and overlap bias associated with other methods of control selection [16,17].

There are several limitations to our study. First, the study population is subject to varying levels of selection bias that may limit the generalizability of the results. Being a clinical trial population, patients with a poor prognosis due to massive hematoma or deep coma, and in whom early surgery was planned, were excluded. Therefore, future studies on a general ICH population are necessary to validate our findings. Another issue is that we were unable to obtain (≤100km) hourly temperature data for 30% of participants (‘excluded patients’), of which the majority were from second to third tier cities in China (Baotou, Xuzhou, Wenzhou and Xining). Differences in the provision of healthcare and medical development, and diet and lifestyles, may have resulted in significant differences in characteristics such as ‘time from onset to randomization’ and use of medications (e.g. antihypertensives and antithrombotics) and contribute to variability in severity of hospital presentation and prognosis, thus limiting the generalizability of our findings. However, sensitivity analyses that included approximated temperature of Baotou (83%) showed a slight increase in ICH risk in association with low temperature in comparison to the primary analyses. A further issue relates to the precision of the exposure temperature data, as some of our weather data for the main analyses originated from monitoring stations up to 100 kilometres away from participating hospitals. However, this may likely have generated a non-differential misclassification that biased the result toward the null, and therefore produced conservative estimates of the true risk. We were also unable to account for potential confounding from individual changes in behaviour and cold protection. We may have been able to partly mitigate this problem through use of the case-crossover design and control selection strategy as the pattern of activity and clothing habits of individuals are likely to remain consistent at across times of the day, days of the week, and within a one-month calendar period. It is also interesting to note that a Eurowinter group study reported no associations between indoor heating, clothing protection, and even sweating, with reductions in cerebrovascular disease related mortality [28]. While we were unable to collect information on other potential confounders, such as the patients’ emotional state that could have a bearing on BP variability, the presence of viral infections, usage of sympathomimetics and of other weather parameters or air pollution, these are also assumed to be constant in case-crossover analysis. Finally, as the study was post-hoc, we cannot exclude the play of chance and reliably establish a causal relationship between low ambient temperature and ICH occurrence.

In conclusion, the present findings based on 1997 patients from a large global study show an increased risk of ICH within a few hours of exposure to very low ambient temperature. The risk of ICH in high-risk subjects might be reduced by more stringent monitoring and management of BP levels during cold seasons, targeted personal advice and environmental heating interventions, triggered by forecasts of very low temperature.

## Supporting Information

S1 Fig Overall cumulative odds ratio plot for temperature effects using approximated weather data. CI indicates confidence interval.
(TIFF)

S2 Fig Overall cumulative odds ratio plot for temperature effects with extended 72h lag period. CI indicates confidence interval.
(TIFF)

S3 Fig Lag-exposure plots of intracerebral hemorrhage for specific temperatures with extended 72h lag period. CI indicates confidence interval.
(TIFF)

S1 Table Weather characteristics of all included INTERACT2 cities. (DOCX)
