# Supplementary figures and images for: Low Ambient Temperature and Intracerebral Hemorrhage: The INTERACT2 Study
Source: PLoS One. 2016 Feb 9;11(2):e0149040. doi: 10.1371/journal.pone.0149040 (PMC4747478; doi:10.1371/journal.pone.0149040)

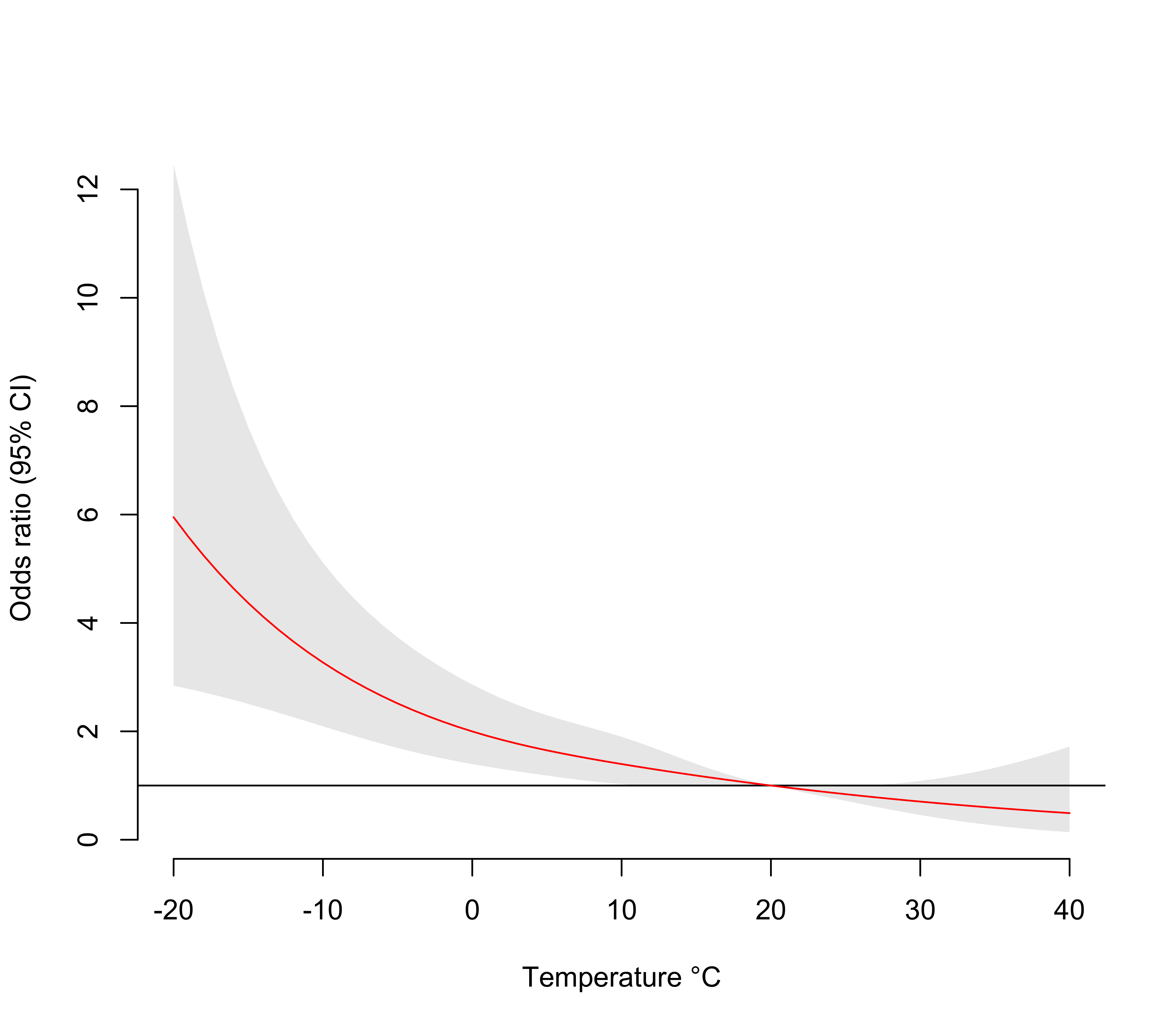

Supplement: S1 Fig — CI indicates confidence interval. (TIFF) [file pone.0149040.s001.tiff]

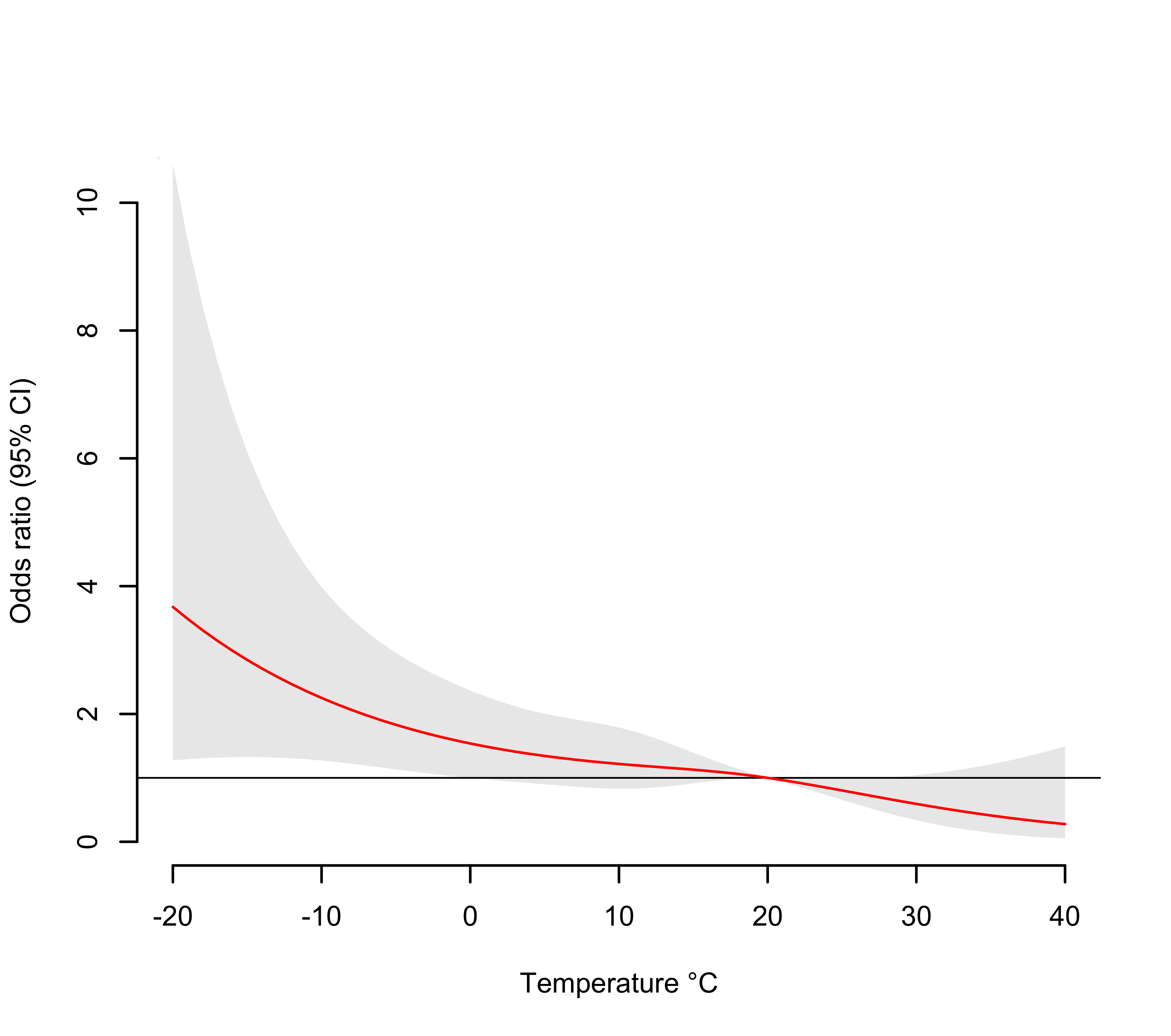

Supplement: S2 Fig — CI indicates confidence interval. (TIFF) [file pone.0149040.s002.tiff]

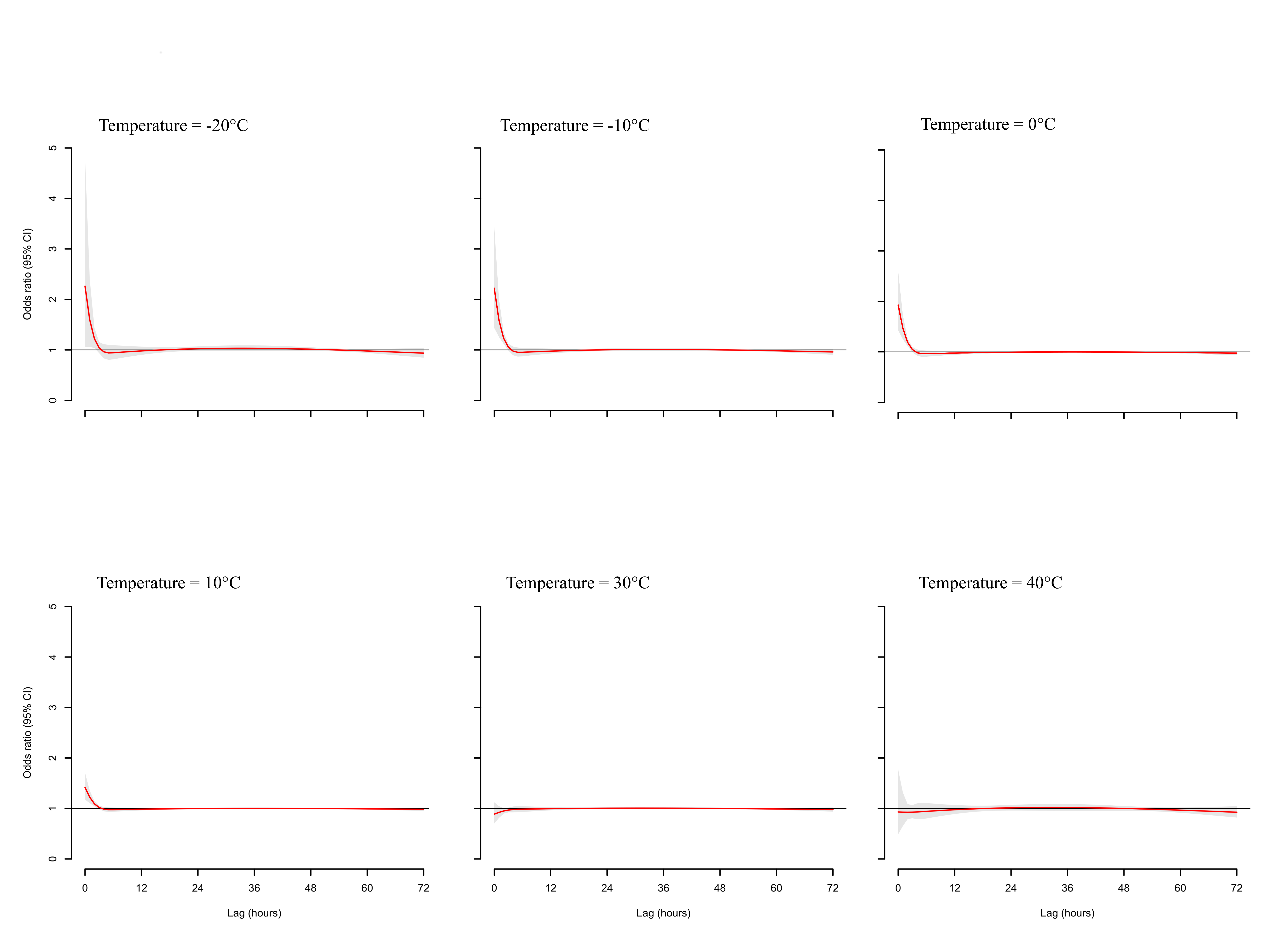

Supplement: S3 Fig — CI indicates confidence interval. (TIFF) [file pone.0149040.s003.tiff]
