# Supplementary material for: Low Ambient Temperature and Intracerebral Hemorrhage: The INTERACT2 Study
Source: PLoS One. 2016 Feb 9;11(2):e0149040. doi: 10.1371/journal.pone.0149040 (PMC4747478; doi:10.1371/journal.pone.0149040)
Supplement: S1 Table — (DOCX) [file pone.0149040.s004.docx]

**Supplementary Table S1. Weather characteristics of all included INTERACT2 cities**

|  | **Patient**  **number** | **Temperature characteristics during study period (°C)^a^** | | | | | |
| --- | --- | --- | --- | --- | --- | --- | --- |
| **Country/City** | **N (%)** | **Mean (SD)** | **Median** | **Min** | **Max** | **25%** | **75%** |
| **Argentina** |  |  |  |  |  |  |  |
| Adrogue | 1 (0.1) | 17 (7) | 17 | -4 | 39 | 12 | 22 |
| Mendoza | 3 (0.2) | 18 (8) | 18 | -5 | 43 | 12 | 24 |
| **Australia** |  |  |  |  |  |  |  |
| Brisbane | 3 (0.2) | 20 (5) | 21 | 4 | 39 | 17 | 24 |
| Gosford | 21 (1.1) | 16 (6) | 17 | -1 | 41 | 12 | 21 |
| Newcastle | 10 (0.5) | 17 (6) | 18 | 0 | 42 | 13 | 21 |
| Sydney | 4 (0.2) | 18 (5) | 18 | 4 | 41 | 15 | 22 |
| Melbourne | 30 (1.5) | 16 (6) | 15 | 2 | 44 | 12 | 19 |
| Perth | 3 (0.2) | 19 (6) | 18 | 3 | 48 | 15 | 22 |
| **Austria** |  |  |  |  |  |  |  |
| Linz | 9 (0.5) | 10 (9) | 10 | -19 | 37 | 3 | 17 |
| Innsbruck | 34 (1.7) | 9 (9) | 9 | -18 | 35 | 2 | 16 |
| Graz | 20 (1.0) | 10 (9) | 11 | -18 | 35 | 3 | 18 |
| **Belgium** |  |  |  |  |  |  |  |
| Brussels | 12 (0.6) | 11 (7) | 10 | -14 | 34 | 6 | 15 |
| **Brazil** |  |  |  |  |  |  |  |
| Rio de Janeiro | 1 (0.1) | 24 (4) | 24 | 11 | 40 | 21 | 27 |
| Sao Paulo | 10 (0.5) | 20 (5) | 20 | 3 | 36 | 17 | 23 |
| Curitiba | 1 (0.1) | 18 (5) | 17 | -1 | 35 | 14 | 21 |
| Porto Alegre | 5 (0.3) | 19 (6) | 19 | -3 | 39 | 15 | 23 |
| **Chile** |  |  |  |  |  |  |  |
| Santiago | 17 (0.9) | 15 (7) | 14 | -4 | 36 | 10 | 20 |
| Los Angeles | 12 (0.6) | 12 (6) | 11 | -5 | 37 | 8 | 15 |
| **China** |  |  |  |  |  |  |  |
| Changchun | 73 (3.7) | 5 (15) | 7 | -34 | 37 | -8 | 19 |
| Beijing | 280 (14.0) | 12 (12) | 14 | -19 | 41 | 1 | 23 |
| Tangshan | 225 (11.3) | 12 (12) | 14 | -19 | 41 | 1 | 23 |
| Cangzhou | 158 (7.9) | 13 (12) | 14 | -18 | 39 | 2 | 23 |
| Shijiazhuang | 83 (4.2) | 11 (12) | 12 | -18 | 40 | 1 | 20 |
| Tianjin | 53 (2.7) | 13 (12) | 14 | -18 | 39 | 2 | 23 |
| Suzhou | 41 (2.1) | 17 (10) | 18 | -6 | 39 | 9 | 25 |
| Shanghai | 93 (4.7) | 17 (10) | 18 | -6 | 39 | 9 | 25 |
| Nanjing | 18 (0.9) | 16 (10) | 17 | -11 | 39 | 7 | 25 |
| Wuxi | 4 (0.2) | 16 (10) | 17 | -11 | 39 | 7 | 25 |
| Changsha | 71 (3.6) | 18 (10) | 19 | -6 | 41 | 9 | 26 |
| Fuzhou | 12 (0.6) | 20 (7) | 21 | 1 | 36 | 14 | 27 |
| Zengcheng | 15 (0.8) | 23 (7) | 25 | 2 | 40 | 18 | 28 |
| Guangzhou | 38 (1.9) | 23 (7) | 25 | 2 | 40 | 18 | 28 |
| Zhuhai | 26 (1.3) | 24 (6) | 25 | 5 | 36 | 20 | 29 |
| Hong Kong (SAR) | 1 (0.1) | 24 (6) | 25 | 5 | 36 | 20 | 29 |
| **Finland** |  |  |  |  |  |  |  |
| Helsinki | 36 (1.8) | 6 (10) | 6 | -31 | 34 | 0 | 13 |
| **France** |  |  |  |  |  |  |  |
| Lille | 30 (1.5) | 11 (7) | 11 | -12 | 35 | 6 | 16 |
| Calais | 12 (0.6) | 11 (6) | 11 | -10 | 32 | 7 | 15 |
| Paris | 143 (7.2) | 12 (7) | 12 | -12 | 37 | 7 | 17 |
| Le Chesnay | 16 (0.8) | 11 (7) | 11 | -13 | 37 | 6 | 16 |
| Blain | 13 (0.7) | 12 (7) | 12 | -8 | 36 | 8 | 17 |

^a^From 01/01/2008 to 01/01/2013

**Supplementary Table S1. Weather characteristics of all included INTERACT2 cities** ***(continued)***

|  | **Patient**  **number** | **Temperature characteristics during study period (°C)^a^** | | | | | |
| --- | --- | --- | --- | --- | --- | --- | --- |
| **Country/City** | **N (%)** | **Mean (SD)** | **Median** | **Min** | **Max** | **25%** | **75%** |
| **Germany** |  |  |  |  |  |  |  |
| Hamburg | 2 (0.1) | 10 (8) | 10 | -17 | 34 | 4 | 15 |
| Berlin | 45 (2.3) | 10 (8) | 10 | -19 | 37 | 4 | 16 |
| Halle | 21 (1.1) | 10 (9) | 10 | -21 | 37 | 4 | 16 |
| Dresden | 27 (1.4) | 10 (9) | 10 | -20 | 37 | 4 | 16 |
| Dusseldorf | 3 (0.2) | 11 (7) | 11 | -14 | 37 | 6 | 16 |
| Frankfurt | 11 (0.6) | 11 (8) | 11 | -16 | 36 | 5 | 17 |
| Mannheim | 23 (1.2) | 12 (8) | 11 | -15 | 37 | 6 | 18 |
| Ulm | 7 (0.4) | 9 (8) | 9 | -19 | 33 | 2 | 15 |
| **India** |  |  |  |  |  |  |  |
| Hyderabad | 10 (0.5) | 23 (9) | 24 | -5 | 43 | 17 | 29 |
| Chennai | 1 (0.1) | 28 (4) | 28 | 17 | 42 | 26 | 31 |
| **Italy** |  |  |  |  |  |  |  |
| Modena | 15 (0.8) | 14 (9) | 14 | -15 | 38 | 6 | 21 |
| Perugia | 22 (1.1) | 14 (9) | 14 | -9 | 40 | 8 | 20 |
| Rome | 10 (0.5) | 16 (8) | 16 | -6 | 39 | 10 | 22 |
| **Netherlands** |  |  |  |  |  |  |  |
| Utrecht | 2 (0.1) | 11 (7) | 11 | -16 | 34 | 6 | 16 |
| **Norway** |  |  |  |  |  |  |  |
| Tromso | 1 (0.1) | 3 (6) | 3 | -17 | 29 | -1 | 8 |
| Lillehammer | 2 (0.1) | 2 (9) | 2 | -33 | 28 | -4 | 9 |
| Kristiansand | 2 (0.1) | 7 (8) | 8 | -23 | 28 | 2 | 13 |
| **Pakistan** |  |  |  |  |  |  |  |
| Karachi | 9 (0.5) | 27 (6) | 28 | 2 | 44 | 24 | 30 |
| **Portugal** |  |  |  |  |  |  |  |
| Oporto | 22 (1.1) | 15 (5) | 15 | -3 | 38 | 12 | 18 |
| **Spain** |  |  |  |  |  |  |  |
| Girona | 34 (1.7) | 15 (8) | 14 | -8 | 37 | 9 | 20 |
| Barcelona | 10 (0.5) | 17 (6) | 16 | -1 | 34 | 12 | 22 |
| **United Kingdom** |  |  |  |  |  |  |  |
| Ashington | 2 (0.1) | 9 (6) | 9 | -11 | 27 | 5 | 13 |
| Newcastle upon Tyne | 8 (0.4) | 9 (6) | 9 | -11 | 27 | 5 | 13 |
| Durham | 1 (0.1) | 9 (6) | 9 | -11 | 27 | 5 | 13 |
| Leeds | 2 (0.1) | 9 (6) | 9 | -12 | 27 | 5 | 13 |
| Salford | 12 (0.6) | 10 (5) | 10 | -12 | 29 | 7 | 14 |
| Nottingham | 2 (0.1) | 10 (6) | 10 | -13 | 29 | 6 | 14 |
| Stafford | 2 (0.1) | 10 (6) | 10 | -19 | 28 | 6 | 14 |
| Wolverhampton | 4 (0.2) | 10 (6) | 10 | -19 | 28 | 6 | 14 |
| Leicester | 3 (0.2) | 10 (6) | 10 | -13 | 29 | 6 | 14 |
| Cambridge | 1 (0.1) | 10 (6) | 11 | -11 | 31 | 6 | 15 |
| Oxford | 3 (0.2) | 10 (6) | 10 | -17 | 30 | 6 | 15 |
| Bristol | 1 (0.1) | 10 (6) | 10 | -8 | 28 | 6 | 14 |
| Bath | 6 (0.3) | 10 (6) | 10 | -8 | 28 | 6 | 14 |
| London | 16 (0.8) | 12 (6) | 12 | -9 | 32 | 7 | 16 |
| Taunton | 1 (0.1) | 10 (6) | 11 | -15 | 28 | 7 | 15 |
| Exeter | 3 (0.2) | 10 (6) | 11 | -15 | 28 | 7 | 15 |
| Bournemouth | 3 (0.2) | 11 (6) | 11 | -11 | 29 | 7 | 15 |
| **USA** |  |  |  |  |  |  |  |
| Rochester | 11 (0.6) | 7 (13) | 8 | -32 | 38 | -2 | 18 |
| **Total** | 1997 (100) | 13 (9) | 13 | -34 | 48 | 7 | 19 |

^a^From 01/01/2008 to 01/01/2013
